# Evidence on percutaneous radiofrequency and microwave ablation for liver metastases over the last decade

**DOI:** 10.1007/s11604-022-01335-5

**Published:** 2022-09-13

**Authors:** Koji Tomita, Yusuke Matsui, Mayu Uka, Noriyuki Umakoshi, Takahiro Kawabata, Kazuaki Munetomo, Shoma Nagata, Toshihiro Iguchi, Takao Hiraki

**Affiliations:** 1 Department of Radiology, Okayama University Hospital, 2-5-1 Shikata-cho, Kita-ku, Okayama, 700-8558 Japan; 2 Department of Radiology, Faculty of Medicine, Dentistry and Pharmaceutical Sciences, Okayama University, 2-5-1 Shikata-cho, Kita-ku, Okayama, 700-8558 Japan; 3 Department of Radiological Technology, Faculty of Health Sciences, Okayama University, 2-5-1 Shikata-cho, Kita-ku, Okayama, 700-8558 Japan

**Keywords:** Ablation, Liver, Metastasis

## Abstract

**Purpose:**

This review aimed to summarize the treatment outcomes of percutaneous radiofrequency ablation (RFA) and microwave ablation (MWA) for metastatic liver tumors based on the findings of published studies over the last decade.

**Materials and methods:**

Literature describing the survival outcomes of ablation therapy for liver metastases was explored using the PubMed database on April 26, 2022, and articles published in 2012 or later were selected. The included studies met the following criteria: (i) English literature, (ii) original clinical studies, and (iii) literature describing overall survival (OS) of thermal ablation for metastatic liver tumors. All case reports and cohort studies with fewer than 20 patients and those that evaluated ablation for palliative purposes were excluded.

**Results:**

RFA was the most commonly used method for ablation, while MWA was used in several recent studies. RFA and MWA for liver metastases from various primary tumors have been reported; however, majority of the studies focused on colorectal cancer. The local control rate by RFA and MWA varied widely among the studies, ranging approximately 50–90%. Five-year survival rates of 20–60% have been reported following ablation for colorectal liver metastases by a number of studies, and several reports of 10-year survival rates were also noted.

**Conclusion:**

Comparative studies of local therapies for colorectal liver metastases demonstrated that RFA provides comparable survival outcomes to surgical metastasectomy and stereotactic body radiation therapy.

## Introduction

Metastases from various primary tumors commonly occur in the liver. Among the 2.4 million patients diagnosed with cancer in the United States from 2010 to 2015, 5% had liver metastases at the time of diagnosis [1]. The 1-year survival rates were reportedly 15.1% and 24.0% in the patients with and without liver metastases, respectively [1]. In general, surgical resection of the liver metastases of colorectal cancer (CRLM) is widely known as a curative treatment and is still considered the gold standard [2]. On the other hand, since only 15–20% of the patients with CRLM are considered candidates for hepatectomy, minimally invasive ablation therapies, such as radiofrequency ablation (RFA) and microwave ablation (MWA), are widely used for unresectable liver metastases, and their efficacy has been investigated in numerous studies, especially in the past decade with an increasing number of publications on MWA [2, 3]. MWA could be employed for ablation over RFA in the future since it provides a large ablation area with short procedural time. This review aimed to summarize the current data on the oncologic outcomes of percutaneous RFA and MWA for liver metastases based on the findings of studies published during or after 2012.

## Literature search

We searched the literature published during or after 2012 with the following terms in the title using the PubMed database on April 26, 2022: (“liver”) AND (“metastasis” OR “metastases” OR “metastatic”) AND (“ablation” OR “ablative”) NOT (“surgery” OR “primary” OR “resection” OR “hepatectomy” OR “radiotherapy”).

## Inclusion criteria

An initial search identified 279 articles. Among them, we scrutinized the titles, abstracts, and texts to identify relevant articles. The inclusion criteria for the studies were: (i) English literature, (ii) original clinical studies, and (iii) literature describing the overall survival (OS) of thermal ablation for metastatic liver tumors.

## Exclusion criteria

All the case reports and cohort studies with fewer than 20 patients, and those that evaluated ablation for palliative purposes were excluded. Reports describing laparoscopic approaches were also excluded.

## Literature screening

**Fig. 1:**
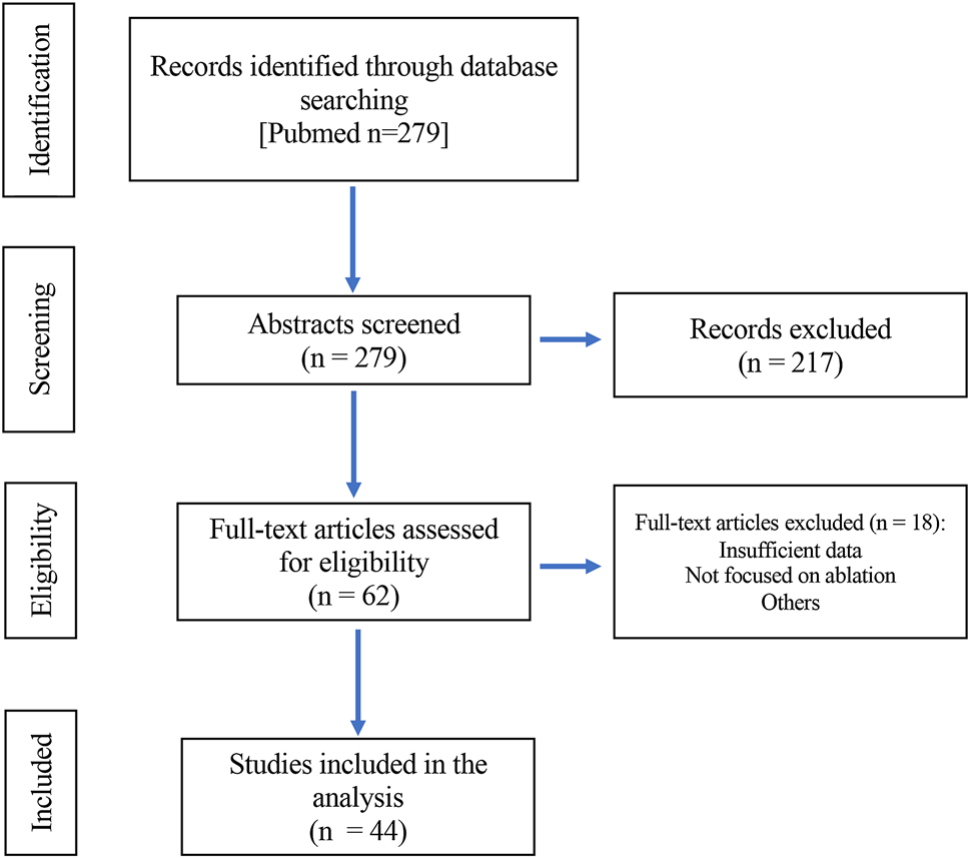
Flowchars of the studies retrieved from the literature search and included in the analysis

Abstracts of the articles were reviewed by two independent investigators (K.T. and M.U.) according to the pre-defined criteria. Investigators then reviewed the entire text of the included articles and extracted the data. An initial search identified 279 articles, of which 62 complete text articles were assessed and 44 were included (Fig. 1).

## Outcomes of thermal ablation for liver metastases arising from various primary tumors.

Table 1 summarize the outcome of ablative therapies for liver metastases from mixed types of primary tumors [4–6]. Vogl et al. conducted a prospective study comparing the oncological outcome and safety of RFA and MWA for liver metastases arising from colorectal, breast, and pancreatic cancers, and reported 2-year OS rates of 62.5% for RFA and 76.9% for MWA [4]. Liu et al. reported the outcomes of 22 patients treated with RFA for metastatic liver cancer arising from the gastrointestinal tract, kidney, lung, etc.; the local control rate was 90.9% with 1-, 3-, and 5-year OS rates of 73.9%, 45.4%, and 37.5%, respectively [5]. Wu et al. reported the outcomes of RFA assisted by contrast-enhanced ultrasonography (CEUS) for 136 patients with 219 liver metastasis arising from colorectal, breast, stomach, lung, esophageal, and pancreatic cancers; the 1-, 2-, and 3-year OS rates were 82.5%, 64.3%, and 50.1%, respectively [6]. In the group of 216 tumors in 126 patients without CEUS, the 1-, 2-, and 3-year OS rates were 73.5%, 44.9%, and 25.3%, respectively [6]. The authors stated that compared to unenhanced ultrasonography (US), CEUS provided better local control since it allowed the exact size of the tumor to be confirmed. Additionally, they suggested that CEUS permits the detection and ablation of new, smaller metastases [6].

**Table 1:** Studies on ablation therapy for liver metastasis from mixed types of primary tumors

Author	Publication year	Type of primary tumor	Type of ablation	No. of patients	Size of tumors [mm]	Follow-up [months]	Local tumor control [%]	Overall survival [months]	Overall survival [%] (Progression-free survival[%])				
									1-year	2-year	3-year	4-year	5-year
Vogl et al. [4]	2022	CRC, breast, pancreas, etc	MWA	26	17.2 (mean)	24	100	–	84.6	76.9	–	–	–
			RFA	24	15.3 (mean)		91.7	–	87.5	62.5	–	–	–
Liu et al. [5]	2014	GI, kidney, lung, etc	RFA	22	–	–	90.9	–	73.9	–	45.4	–	37.5
Wu et al. [6]	2013	CRC, breast, stomach, etc	RFA	136 (CEUS group)	32 (mean)	16.3	83	38	82.5	64.3	50.1 (38.3)	–	–
				126 (control group)	34 (mean)	21.9	70.6	20	73.5	44.9	25.3 (19.3)	–	–

*RFA* radiofrequency ablation, *MWA* microwave ablation, *CRC* colorectal cancer, *GI* gastrointestinal

## Results in each type of primary tumor

### Colorectal cancer

While the majority of the articles were of retrospective studies, Yamakado et al. presented a prospective, multicenter study of RFA with hepatic arterial chemoembolization for CRLM [7]. Their study included 25 patients with 38 tumors; the reported 2-year local control rate was 94.6% on tumor basis and the 2-year overall and recurrence-free survival rates were 88.0% and 63. 3%, respectively [7]. A number of studies have reported the oncologic outcomes of ablative therapies for CRLM describing the 5-year OS, and even 10-year or longer survival rates have been mentioned in some articles. Solbiati et al. retrospectively evaluated the outcome of RFA for CRLM; the 1-, 3-, 5-, 7-, and 10-year OS rates were 98%, 69.3%, 47.8%, 25.0%, and 18.0%, respectively, with a median follow-up of 72 months [8]. Han et al. reported 1-, 5-, 10-, and 15-year OS rates of 92%, 41%, 30%, and 28% following RFA for CRLM [9]. Jiang et al. retrospectively evaluated the outcomes of RFA in patients with CRLM; the 1-, 3-, 5-, and 10-year OS rates in perivascular/non-perivascular tumor groups were 91.3/85.0, 45.6/51.9, 23.9/25.6, and 18.7/22.6%, respectively [10]. Cheng et al. reported the outcome of RFA using a multielectrode radiofrequency switching controller for resectable metachronous CRLM; the 5-year survival and median OS were 50.7% and 53.4 months, respectively [11]. Table 2 presents the outcomes of RFA and MWA for CRLM including the 5-year OS rates [8–27]. In the listed studies, 5-year OS following ablation ranged from 21 to 65%.

**Table 2:** Studies on ablation therapy for liver metastasis from colorectal cancer

Author	Publication year	Type of ablation	No. of patients	No. of tumors	Size of Tumors [mm]	Follow-up [months]	Local tumor control [%]	Overall survival [months]	Overall survival [%] (Disease- or Progression-free survival [%])				
									1-year	2-year	3-year	4-year	5-year
Cheng et al. [11]	2021	RFA	68	–	29 (median)	59.8 (median)	47.1	53.4 (median)	88.1	–	66.9	–	64.5
Knott et al. [12]	2021	MWA	57	102	18 (mean)	42 (median)	96	52 (median)	96 (93)	–	66 (58)	–	47 (39)
Fan et al. [13]	2021	RFA	199	402	16 (mean)	23 (median)	62.9	Subcapsular 46 (median)	96.1	–	66	–	44.2
								non-subcapsular 60 (median)					
Kurilova et al. [14]	2021	RFA^a^ MWA	286	415	–	31 (median)	–	41.5 (median)	91 (67)	72 (55)	53 (47)	–	37
Han et al. [9]	2021	RFA	365	512	–	43.1(median)	75.4	44 (median)	92 (85)	–	58 (74)	–	41 (73)
Wang et al. [15]	2020	RFA	85	138	28 (mean)	30 (median)	67.4	36 (median)	90.6	–	45.6	–	22.9
Thai et al. [16]	2020	RFA	61	166	–	24 (median)	88.5	32 (median)	93.2	–	44.5	–	38.2
Wang et al. [17]	2020	RFA	81	126	25(mean)	51.2 (median)	52.8	26.8 (median)	81.2 (59.3)	(51.4)	32.1 (46.6)	–	23.9
Jiang et al. [10]	2020	RFA	104 perivascular group	388	24 (mean)	45.0 (median)	82.7	–	91.3 (85.2)	–	45.6 (81.1)	–	23.9 (81.1)
			284 non-perivascular group				85.2	–	85 (88.8)	–	51.9 (80.3)	–	25.6 (78.6)
Mao et al. [18]	2019	RFA	61	114	27 (median)	28.9 (median)	83.3	–	–	–	(70.9)	–	33
Ou et al. [19]	2018	RFA	109	109	34 (mean)	26 (median)	–	56.5 (mean)	92.3	–	50.7	–	41.6
Shady et al. [20]	2017	RFA	97	–	17 (median)	60.1 (median)	56.1	35.5 (median)	89.6 (68.8)	–	48.5 (50.1)	–	30.3 (47.3)
Facciorusso et al. [21]	2016	RFA	127	193	27 (median)	63 (median)	79.5	38 (median)	89.4	–	–	40.4	33.3
Shady et al. [22]	2016	RFA	162	233	18 (median)	55 (median)	52	36 (median)	90	–	48	–	31
Facciorusso et al. [23]	2016	RFA	143	–	26 (median)	72 (median)	81.1	44 (median)	91.4	–	–	46.5	42.2
Zhang et al. [24]	2016	MWA	199	318	30 (median)	30.2 (median)	94.9	41 (median)	–	–	–	–	27.9 (10.1)
Labori et al. [25]	2015	RFA	52	70	–	–	–	36 (median)	–	–	–	–	27
Hamada et al. [26]	2012	RFA	84	141	23(mean)	27.0 (median)	72.3	34.9 (median)	90.6 (73.7)	–	44.9 (55.1)	–	20.8 (47.2)
Solbiati et al. [8]	2012	RFA	99	202	22 (mean)	72 (median)	67.7	53 (median)	98	–	69.3	–	47.8
Veltri et al. [27]	2012	RFA	248	458	–	26.4 (mean)	–	32 (median)	84	59	43	–	23

^a^RFA and MWA were used for 56% and 44% of sessions, respectively

*RFA* radiofrequency ablation, *MWA* microwave ablation

In these studies, the prognostic factors for ablation treatment of liver metastases have also been investigated. Several studies reported that a tumor diameter of ≤ 30 mm [10, 15–17, 21–23, 26] or ≤ 40 mm [24] was a positive prognostic factor following ablation. In addition, complete ablation of the metastatic liver tumor [19] and re-treatment of local tumor progression (LTP) [8] contributed to a favorable prognosis. Zhang et al. demonstrated that patients who received chemotherapy of more than six cycles following RFA demonstrated a longer OS [24]. Stang et al. reported that patients who met the four criteria (effective chemotherapy before RFA, tumor diameter ≤ 3 cm, tumor number ≤ 3, and carcinoembryonic antigen ≤ 100 ng/ml) showed significantly better 5-year survival and recurrence-free survival rates of 39 and 22%, respectively, than those patients who did not meet the criteria [28]. The other favorable prognostic factors were 25-hydroxyvitamin D > 20 ng/mL [23] and lymphocyte-to-monocyte ratio > 3.96% [21]. Several studies have demonstrated a larger number of liver metastasis [10, 15, 16, 26] and the presence of extrahepatic metastases [10, 15, 18, 20, 22, 26] to be associated with a significantly shorter OS. The other reported negative prognostic factors were *Kras* mutation [20], *BRAF* mutation [11], intrahepatic recurrence [10], LTP at the 6-month follow-up [17], high tumor grade [11], distant metastasis at diagnosis of the primary tumor [11], and previous radiation therapy [11].

Local tumor control rate of RFA for CRLM ranges from 47 to 96%, which is inferior to RFA for early-stage hepatocellular carcinoma (86–99%) [3]. Kurilova et al. reported a significantly higher LTP free survival with a larger minimal ablation margin [14]. LTP rate was 0% at > 10 mm, 26% at 6–10 mm, 60% at 1–5 mm, and 79% at no margin or 0 mm, respectively [14]. Wu et al. reported that 47.0% (63/134) of the metastatic liver tumors examined with CEUS were larger than 0.3 cm compared to the unenhanced US, and 90.5% of these tumors were larger than 2 cm [6]. Variation in local control among the studies could be caused by a tendency for RFA for liver metastases to have small ablation margins.

### Breast cancer

Table 3 summarize the outcome of the ablative therapies for liver metastases from breast cancer [29–32]. Schullian et al. reported the outcomes of RFA in 42 patients with 110 liver metastases [29]. They demonstrated a local control rate of 92.7% and 1-, 3-, and 5-year OS rates of 84.1%, 49.3%, and 20.8% respectively [29]. Bai et al. also reported similar results of RFA for liver metastases, with a local control rate of 88.4% and 1-, 2-, 3-, and 5-year survival rates of 81.8%, 50.1%, 25.3%, and 11%, respectively [30]. Age > 60 years [29], tumor size [29], positive estrogen receptor status [30], and extrahepatic metastatic disease were reported as unfavorable prognostic factors [29, 30].

**Table 3:** Studies on ablation therapy for liver metastasis from breast cancer

Author	Publication year	Type of ablation	No. of patients	No. of tumors	Size of tumors [mm]	Follow-up [months]	Local tumor control [%]	Overall survival [months]	Overall survival [%]				
									1-year	2-year	3-year	4-year	5-year
Schullian et al. [29]	2021	RFA	42	110	30 (median)	10.9 (median)	92.7	32.3 (median)	84.1	–	49.3	–	20.8
Bai et al. [30]	2019	RFA	69	135	29 (median)	26 (median)	88.4	26 (median)	81.8	50.1	25.3	–	11
Bale et al. [31]	2018	RFA	26	64	28 (median)	23.1 (median)	92.2	29.3 (median)	–	–	–	–	–
Kümler et al. [32]	2015	RFA	32	–	20 (median)	–	78	33.5 (median)	87	68	48	–	–

*RFA* radiofrequency ablation

### Gastric cancer

Ablative therapies for liver metastases from gastric cancer are summarized in Table 4 [33–36]. RFA was always employed in the studies except for one by Zhou et al. in which MWA was employed [34]. The 1-, 3-, and 5-year OS rates were reportedly 59–81%, 5–31%, and 3–16.7%, respectively. All the studies reported a negative prognosis for multiple metastases compared to solitary metastasis [33–36]. The other negative prognostic factors shown in the studies included extra hepatic metastases [34, 35], absence of chemotherapy [35], larger liver metastases [33, 34], and poorly differentiated tumor [33].

**Table 4:** Studies on ablation therapy for liver metastasis from gastric cancer

Author	Publication year	Type of ablation	No. of patients	No. of tumors	Size of tumors [mm]	Follow-up [months]	Local tumor control [%]	Overall survival [months]	Overall survival [%]				
									1-year	2-year	3-year	4-year	5-year
Fan et al. [33]	2020	RFA	46	–	–	24.9 (median)	–	26.1 (median)	58.7	23.9	–	–	–
Zhou et al. [34]	2017	MWA	32	–	38 (mean)	19 (median)	100	25 (median)	80.9	–	31.2	–	16.7
Hwang et al. [35]	2014	RFA	44	–	20 (median)	31.7 (median)	–	14.7 (median)	–	–	–	–	–
Chen et al. [36]	2013	RFA	21	32	38 (median)	19 (median)	81	14 (median)	70	11	5	–	3

*RFA* radiofrequency ablation, *MWA* microwave ablation

### Pancreatic ductal adenocarcinoma

Two studies reported on ablation for liver metastases arising from pancreatic cancer are summarized in Table 5, which were relatively new and published in the year 2021 and 2022 [37, 38]. The 1-, 2-, and 3-year OS rates following ablation were reportedly 40–81%, 18–68%, and 53%, respectively, with most patients treated by RFA [37, 38]. Yan et al. treated 104 patients using a combination of ablation and chemotherapy (*n* = 70) or chemotherapy alone (*n* = 30); the 1-, 2-, and 3-year OS rates were 81% and 60%, 68% and 20%, and 53% and 0% in the ablation-chemotherapy and chemotherapy alone groups, respectively [38]. Yan et al. reported a tumor number ≤ 2, complete ablation, and multiple ablation as independent prognostic factors [38]. Du et al. reported that the abnormal serum CA19-9 level and extrahepatic metastases before RFA were independent prognostic factors for OS [37].

**Table 5:** Studies on ablation therapy for liver metastasis from Pancreatic ductal adenocarcinoma

Author	Publication year	Type of ablation	No. of patients	No. of tumors	Size of tumors [mm]	Follow-up [months]	Local tumor control [%]	Overall survival [months]	Overall survival [%] (Progression-free survival [%])				
									1-year	2-year	3-year	4-year	5-year
Du et al. [37]	2022	RFA	20	29	26 (mean)	–	85	14.6 (mean)	39.5 (26.0)	18.1 (17.3)	–	–	–
Yan et al. [38]	2021	RFA/MWA	74	–	–	27.8 (median)	–	10.8 (median)	81.1	67.6	52.6	–	–

*RFA* radiofrequency ablation, *MWA* microwave ablation

### Other tumors

Table 6 summarizes the outcome of ablative therapies for liver metastases from various types of primary tumors [39–42]. Bale et al. reported the result of RFA for liver metastasis from ocular and cutaneous melanoma [39]. In their study, 20 patients with 75 tumors were treated, and the 5-year local control rate was 75% and the 1-, 3- and 5-year OS rates were 64%, 41%, and 17%, respectively [39]. They also reported that the median OS from the date of RFA was 38 months in ocular melanoma and 11.6 months in cutaneous melanoma [39].

**Table 6:** Studies on ablation therapy for liver metastasis from various types of primary tumors

Author	Publication year	Type of primary tumor	Type of ablation	No. of patients	Size of tumors [mm]	Follow-up [months]	Local tumor control [%]	Overall survival [months]	Overall survival [%]				
									1-year	2-year	3-year	4-year	5-year
Wu et al. [42]	2022	Ovarian Cancer	RFA	30	–	–	–	39 (median)	93.3	80	53.3	–	–
Zhao et al. [41]	2020	NSCLC	RFA/MWA	21	24.4 (mean)	36.4 (median)	85.7	27.7 (median)	–	–	–	–	–
Bale et al. [39]	2016	Malignant melanoma	RFA	20	17 (median)	–	86.7	19.3 (median)	64	–	41	–	17
Jin et al. [40]	2012	Nasopharyngeal	RFA	50	–	–	48	14.8 (mean)	56	14	2	–	–
			RFA + CX	44	–	–	63.6	30.6 (mean)	91	61	36	–	–

*RFA* radiofrequency ablation, *MWA* microwave ablation

A study by Jin et al. demonstrated the result of RFA for liver metastasis arising from nasopharyngeal carcinoma [40]. Of the 134 patients, 40, 50, and 44 patients received chemotherapy alone, RFA alone, and RFA and chemotherapy, respectively; the 1-, 2-, and 3-year OS were 56%, 14%, and 2% in the RFA alone group, 60%, 38%, and 13% in the chemotherapy alone group, and 91%, 61%, and 36% in the RFA and chemotherapy group, respectively [40]. No response to treatment, number of liver metastases > 1, liver metastases > 3 cm, disease-free survival (DFS) > 12 months were reported to be significant negative prognostic factors for the OS [40].

Zhao et al. reported the result of RFA for liver metastasis from non-small cell lung cancer [41]. In their study, 21 patients received systemic therapy with RFA and 40 received systemic therapy only [41]. In the systemic therapy with RFA group and systemic therapy alone groups, the median progression-free survival (PFS) was 11.0 and 5.2 months, and the median OS was 27.7 and 17.7 months, respectively [41]. Patients who received systemic therapy with RFA had significantly better PFS; however, no significant difference in the OS was observed [41].

Wu et al. evaluated the outcome of RFA for liver metastasis from ovarian cancer [42]. In their study, the outcomes of RFA with chemotherapy and chemotherapy alone were compared. The 1-, 2-, and 3-year OS rates were 93.3%, 80.0%, and 53.3% in the RFA with chemotherapy group and 79.5%, 60.1%, and 42.1% in the chemotherapy alone group, respectively [42]. Median OS was better in the RFA-chemotherapy group (39.0 months and 34.0 months); however, no statistically significant difference was observed between the two groups [42].

## Effect of ablative therapy in combination with chemotherapy on the survival

In addition to the aforementioned articles [38, 40–42], several authors have compared the survival outcomes between combined therapy of ablation and chemotherapy and chemotherapy alone. Yang et al. conducted a study including CRLM and reported that RFA with chemotherapy prolonged the median OS (29 vs. 12 months, *p* = 0.002) compared to chemotherapy alone [43]. A retrospective study by Kaganov et al. included 176 patients with colorectal cancer with 824 liver metastases; 98 and 78 of whom underwent RFA with chemotherapy and chemotherapy-alone, respectively [44]. In the RFA with chemotherapy and chemotherapy-alone groups, the 1-, 2-, and 3-year OS were 73.5% and 39.6%, 25.1% and 6.3%, and 7.2% and 2.1%, respectively, and the median OS was 18 and 11 months, respectively [44]. The OS was significantly better in the RFA with chemotherapy group than in the chemotherapy-alone group [44]. Ruers et al. reported the results of a randomized prospective comparative trial of RFA with chemotherapy and chemotherapy alone for CRLM in 119 patients [45]. In RFA with chemotherapy and chemotherapy-alone groups, the median OS was 45.3 and 40.5 months (*p* = 0.22), respectively, and the 3-year PFS was 27.6% and 10.6% (*p* = 0.025), respectively [45]. The results of these studies suggest that RFA combined with chemotherapy could contribute to the prolonged survival of patients with CRLM, and supports the incorporation of ablation therapy in the treatment strategy of CRLM when possible.

## Comparison with surgical metastasectomy and radiation therapy

Tago et al. compared the prognosis between 92 patients undergoing surgery for CRLM and 26 patients undergoing RFA for unresectable CRLM [46]. The median OS was 49.5 and 44.9 months in the surgery and RFA groups, respectively, and was significantly shorter in the RFA group [46]. However, this result could be attributed to the different patient backgrounds between the two groups; the number of liver metastases and extrahepatic metastases were significantly larger in the RFA group, and only unresectable tumors were treated by RFA. Cheng et al. compared the outcome of the treatment for resectable metachronous CRLM among three groups of patients who underwent one of following therapies: hepatectomy, RFA with a multielectrode RF switching controller, and systemic treatment only [11]. The 5-year and median OS were 54.9% and 69.8 months in the hepatectomy group, 50.7% and 53.4 months in the RFA group, and 10.2% and 19.1 months in the systemic treatment group, respectively, with a median follow-up of 59.8 months [11]. Both the hepatectomy and RFA groups had significantly longer OS than that in the systemic treatment-only group; moreover, no significant difference was observed in the OS upon the comparison of the hepatectomy and RFA groups [11].

Yu et al. investigated the outcomes of RFA and stereotactic body radiation therapy (SBRT) in 222 patients with 330 CRLM [47]. The RFA and SBRT groups included 178 patients with 268 tumors and 44 patients with 62 tumors, respectively; the mean tumor diameters were significantly smaller in the RFA group (15 mm in the RFA group and 23 mm in the SBRT group) [47]. The 1-, 3-, and 5-year local PFS rates were 72%, 60%, and 58% in the RFA group, and 90%, 78%, and 76% in the SBRT group, respectively; no significant difference was observed between the two groups. Upon analysis of the subgroups with tumor diameter > 2 cm, a significantly better local PFS was found in the SBRT group than in the RFA group [47]. The 1- and 3-year OS rates were 91% and 56% in the RFA group and 96% and 58% in the SBRT group, respectively; no significant difference was observed between the two groups [47].

The results of the above-mentioned studies suggest that the survival outcomes following RFA for CRLM are comparable to those following surgical metastasectomy and SBRT provided that the patient background remains the same. Ablation therapy possesses certain advantages over other treatment modalities. Ablation therapy is often performed in patients who are not amenable to hepatectomy and who are often elderly and in poor general condition. Furthermore, unlike SBRT or surgery, a strong advantage of ablation therapy is that repeated ablation for local progression is possible.

## Conclusion

The oncologic outcomes following RFA and MWA for liver metastases from various types of primary tumors have been investigated in many studies. Studies most frequently focused on CRLM, followed by metastases arising from breast, gastric, and pancreatic cancer. RFA was commonly used for any type of tumor, and MWA was used in several recent studies. The procedures were performed mainly under US guidance, followed by CT guidance. The long-term survival outcomes following ablation for CRLM range relatively widely by the studies, however, they are presumably acceptable. Further accumulation of data is necessary for the ablation of liver metastases arising from other primary tumors. RFA combined with chemotherapy could contribute to the prolonged survival of patients with CRLM. In addition, RFA may provide comparable survival outcomes to surgical metastasectomy and SBRT for patients with CRLM.
